# Vasopressin enhances human preemptive strike in both males and females

**DOI:** 10.1038/s41598-019-45953-y

**Published:** 2019-07-04

**Authors:** Atsushi Kawada, Miho Nagasawa, Aiko Murata, Kazutaka Mogi, Katsumi Watanabe, Takefumi Kikusui, Tatsuya Kameda

**Affiliations:** 1 Department of Social Psychology, The University of Tokyo, Tokyo, Japan; 2 Department of Animal Science and Biotechnology, Azabu University, Kanagawa, Japan; 3 NTT Communication Science Laboratories, NTT Corporation, Kanagawa, Japan; 4 Faculty of Science and Engineering, Waseda University, Tokyo, Japan; 5 Creative Robotics Lab, University of New South Wales, Sydney, Australia; 6 Brain Science Institute, Tamagawa University, Tokyo, Japan; 7 Center for Experimental Research in Social Sciences, Hokkaido University, Hokkaido, Japan

**Keywords:** Human behaviour, Animal behaviour, Social evolution

## Abstract

The neuropeptide arginine vasopressin (AVP), which is known to modulate a wide range of social behaviors in animals, has been identified as a modulator of various negative responses to social stimuli in humans. However, behavioral evidence directly supporting its involvement in human defensive aggression has been rare. We investigated the effect of intranasal AVP on defensive aggression in a laboratory experiment, using an incentivized economic game called the “preemptive strike game” (PSG). Participants played PSG individually (1 on 1) as well as in pairs (2 on 2) under either AVP or saline. We observed that exogenous but not basal AVP modulated the attack rate in PSG for both male and female participants. A model-based analysis of the aggregation of individual attack preferences into pair decisions revealed that the AVP effect on defensive aggression occurred mainly at the individual level and was not amplified at the pair level. Overall, these results present the first evidence that intranasal AVP promotes human defensive aggression for both males and females in a bilateral situation where each party can potentially damage the resources of the other party. These findings also parallel accumulating evidence from non-human animals concerning AVP’s involvement in territorial defense against potential intruders.

## Introduction

Arginine vasopressin (AVP) is a peptide hormone known to act in both central and peripheral organs of animals. Peripherally, it acts as an antidiuretic hormone, modulating water reabsorption in the kidney to maintain water homeostasis. Centrally, it has been reported that AVP is involved in negative or agonistic responses to social stimuli by both human^1–6^ and non-human animals^7,8^. From an evolutionary point of view, it has been argued that the agonistic function of AVP may have resulted from exaptation for some species. Mammals, for example, use urination as a signaling method, including territory marking, and thus the urinary function of AVP may have been adapted to promote defensive aggression against territory intruders^9,10^. At the same time, arginine vasotocin (AVT), a neuropeptide homologous to AVP in non-mammalian vertebrates, is also known to influence various agonistic behaviors including mate competition and territory defense, which suggests that the agonistic functions of AVP/AVT are highly preserved across many taxa, while peptide-behavior relationships are complex and likely to be species-specific^11,12^.

To our knowledge, there have been 20 to 30 experimental studies to date that address the effects of intranasal administration of AVP to humans. These studies are roughly classifiable as examining responses to facial stimuli in non-directly-threatening contexts^3–6,13,14^, responses to social evaluation about self ^2,15,16^ or responses in behavioral games^17–21^. For example, intranasal AVP enhanced agonistic facial-muscular responses to the neutral faces of unfamiliar men among male participants, and enhanced affiliative facial motor patterns to the neutral faces of unfamiliar women among female participants^3,4^. AVP also elevated salivary cortisol among males in the Trier Social Stress Test (TSST), in which the participants had to perform tasks in front of judges^16^. In behavioral games, intranasal administration of AVP increased reciprocation of cooperation in Prisoner’s Dilemma^20^ and risky collaboration among players in Stag Hunt^17^. However, as far as we know, there have been no studies that directly test the effects of AVP on human defensive aggression against potential attacks on resources. The mixed motive games used in previous research (Prisoner’s Dilemma^18–21^ and Stag Hunt^17^) are suited to studying human cooperation and collaboration^22,23^, but their relation to defensive aggression is not necessarily direct.

**Figure 1:**
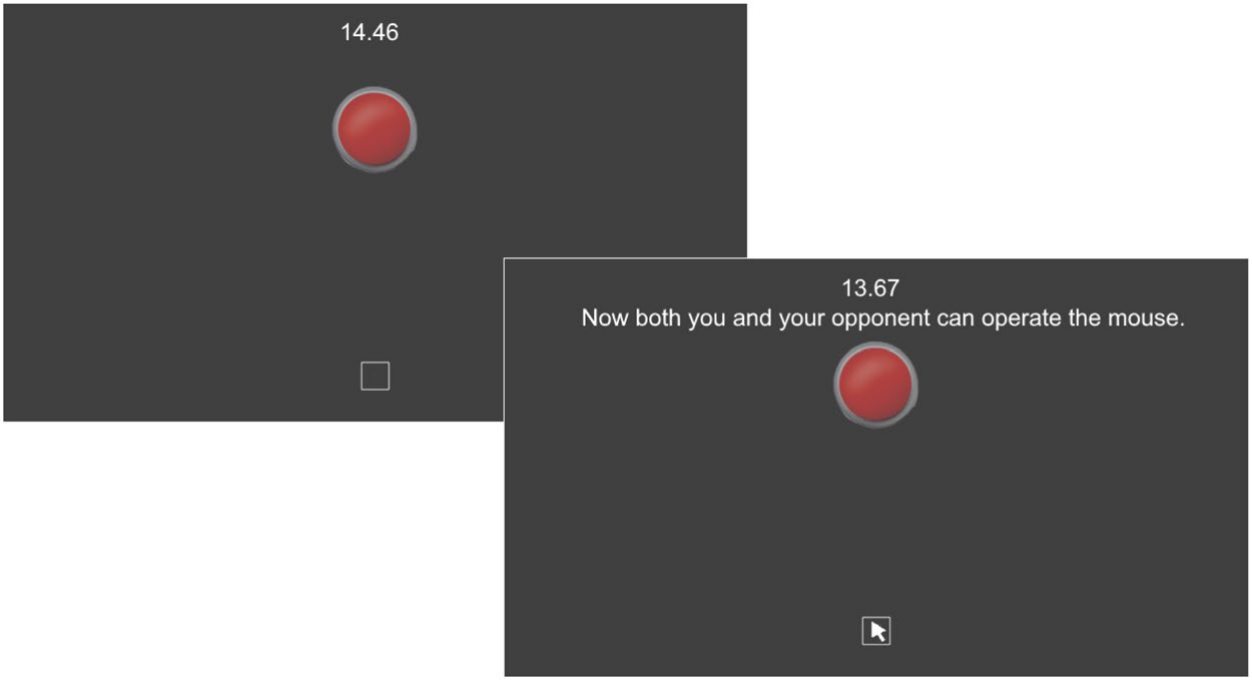
Participant display during the PSG. The remaining time (out of 15 seconds) was displayed at the top. Two to four seconds (randomly jittered) after the game started, a mouse cursor appeared in the white box at the bottom of the computer display. This onset timing was identical for both players. A player who decided to “attack” (though we did not use such loaded words anywhere in the instructions or on the screen) then moved the mouse cursor to the red button and clicked it.

Here, we examined the effects of intranasal AVP on human defensive aggression using a two-person Preemptive Strike Game (PSG)^24^. The PSG captures concern for resource defense in a bilateral interactive context, in which participants decide whether or not to use some of their endowment in order to strike their counterpart preemptively and thus prevent the potential harm of a strike to their own resources (See Fig. 1 and the Method section below for details of our implementation of the game). Previous behavioral studies of PSG reported that the risk of being attacked^25^ and perception of potential threat posed by a counterpart’s group^26^ increased attack rates. Based on the animal literature on territory defense against intruders^9,10^, we expected that intranasal AVP would enhance preemptive strike rates by human participants in PSG as well. Although there have been some reports that AVP’s agonistic function when perceiving neutral faces was greater for males than for females^3,4^, no strong sex difference in cooperative responses in behavioral games has been observed^18–21^. Here we examined both sexes to see if there were any male-female differences in human defensive aggression due to exogenous AVP.

We were also interested in the possibility that the AVP effect, if any, might be amplified at the group level^27^. Because humans have evolved in small, closely-related social groups^28^, AVP may enhance human defensive aggression particularly at the group level. We thus employed both 1-on-1 and 2-on-2 PSG protocols. In both versions, participants played the game against other participants of the same sex (see the Method section).

Participants first played the 1-on-1 PSG against a randomly matched participant. As shown in Fig. 1, participants had 15 seconds to decide whether to take no action or to incur a cost to engage in a preemptive strike (though we did not use such loaded words anywhere in the instructions or on the computer screen). If both players chose to take no action during the 15 seconds, each player could keep the initial endowment intact, receiving 3,000 JPY (about 27 USD). Otherwise, the player who clicked the button first would incur a 700 JPY cost (and thus receive a net 2,300 JPY), and the other player who took no action during the 15 seconds or clicked the button later than their counterpart would lose 2,500 JPY (and thus receive only 500 JPY). Because this “attack” entails a cost to the attacker (700 JPY), a game-theoretically rational strategy in this game is not to attack (i.e., take no action during the 15 seconds), assuming that the other player is also rational.

For analysis, we classified the participants’ behaviors in PSG into one of the three categories:

1. “Attack”: Clicking the red button within the 15 seconds,
2. “Move”: Moving the mouse cursor around the screen but not clicking the red button until the end, or
3. “Untouched”: Leaving the mouse untouched in the white box (Fig. 1) throughout the 15 seconds.

Because never touching the mouse is the surest way to avoid unintended strikes, these 3 categories of behavior are ordered to reflect differential readiness to strike the opponent. We thus treated them as levels of an ordered categorical variable in the following analyses.

## Results

**Figure 2:**
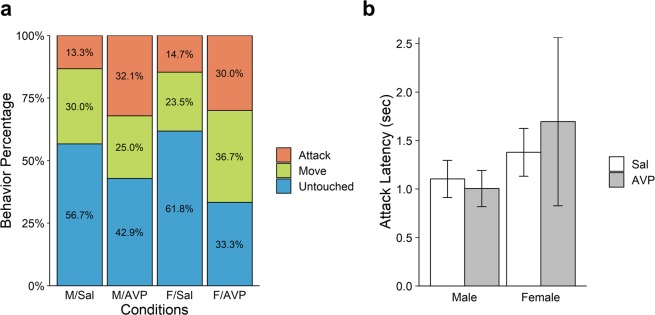
Results of the 1-on-1 PSG. (**a**) Percentage of Attack, Move or Untouched in the 1-on-1 game as a function of Treatment (Sal: saline vs. AVP) and Sex (M: male vs. F: female). “Attack” means clicking the red button during the 15 seconds of the trial. “Move” means moving the mouse cursor but not clicking the red button. “Untouched” means leaving the mouse in its initial position throughout the 15 seconds. (**b**) Mean latencies of attack as a function of Treatment (Saline vs. AVP) and Sex in the 1-on-1 PSG (among the 27 participants who chose Attack in the 1-on-1 PSG). Error bars indicate standard errors of the mean.

Figure 2a displays the percentages of the three types of behavior in the 1-on-1 PSG, as a function of Treatment (Saline vs. AVP) and Sex. Collapsed across both sexes, Attack was more prevalent in the AVP condition (31.0%) than in the Saline condition (14.1%), whereas Untouched was less frequent in the AVP condition (37.9%) than in the Saline condition (59.4%). As these behavioral categories were ordered, we analyzed the data using a proportional-odds ordinal logistic regression model. We first conducted a model selection using the Akaike Information Criterion (AIC) with Treatment (Saline vs. AVP), Sex (Male vs. Female), and the interaction as explanatory variables. As shown in Supplementary Table S1, the model with only a main effect of Treatment was best in terms of AIC. The coefficient for Treatment was positive and significant (*β* = 0.91, *SE* = 0.35, *Z* = 2.62, *p* = 0.010). This result suggests that, for both sexes, AVP enhanced readiness for preemptive strike in the 1-on-1 game.

Figure 2b displays mean latency of attack (in seconds) as a function of Treatment and Sex. We included only the 27 participants who chose Attack in the 1-on-1 PSG in this analysis. As shown in the figure, most attacks occurred early in the trial, within a few seconds after the “attack” button became available to participants (Fig. 1). This quick attack pattern is consistent with results from previous behavioral (non-hormonal) research on PSG^24,27^. A 2 (Treatment) × 2 (Sex) Analysis of Variance (ANOVA) also yielded a main effect of sex, *F* (1, 26) = 6.955, *p* = 0.015. As seen in the figure, mean latencies of attack were longer for females than males, irrespective of the treatment. No other effects were significant.

Without providing any feedback about outcomes from the 1-on-1 session, the experiment proceeded to the 2-on-2 (pair vs. pair) situation. Participants were instructed that they would each be paired with another participant to form a team and play the PSG together against another pair (see the Method section for details).

**Figure 3:**
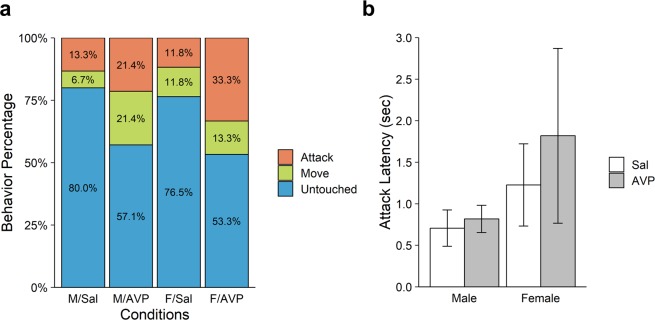
Results of the 2-on-2 PSG. (**a**) Percentage of Attack, Move or Untouched in the 2-on-2 game as a function of Treatment (Saline vs. AVP) and Sex. (**b**) Attack latency as a function of Treatment (Saline vs. AVP) and Sex in the 2-on-2 PSG (among the 12 pairs who chose Attack in the 2-on-2 PSG). Error bars indicate standard errors of the mean.

In the analysis of the 2-on-2 game, we treated each pair of participants as a decision unit, so the total sample size was reduced by half (*N* = 61). Figure 3a displays the percentages of the three types of behavior in the 2-on-2 PSG, as a function of Treatment (saline vs. AVP) and Sex. As in the 1-on-1 game, Attack was more frequent in the AVP condition (27.6%) than in the Saline condition (12.5%), whereas Untouched was less frequent in the AVP (55.2%) than in the Saline condition (78.1%). Again, model selection showed that the proportional-odds ordinal logistic regression model with only a main effect of Treatment was best in terms of AIC (see Supplementary Table S2). The coefficient for Treatment was positive and marginally significant (*β* = 1.05, *SE* = 0.56, *Z* = 1.88, *p* = 0.060), replicating the pattern in the 1-on-1 game, though the effect was weaker at the pair level.

Mean latency of attack in the 2-on-2 PSG revealed essentially the same pattern as in the 1-on-1 PSG. As shown in Fig. 3b, mean latencies were marginally longer for female pairs than male pairs, *F* (1, 11) = 3.744, *p* = 0.089 by A 2 (Treatment) × 2 (Sex) ANOVA. No other effects were significant.

Next we explore how the 2-on-2 game compared to the 1-on-1 game in terms of the likelihood of preemptive strike. We examined possible emergent effects from pairing participants in the 2-on-2 PSG. Because the unit of analysis and, accordingly the sample size, in the 2-on-2 PSG were different from those in the 1-on-1 PSG, it was not possible to simply enter the effect of decision-unit as an additional explanatory variable in the proportional-odds ordinal logistic regression model. We thus conducted the following model-based analysis, in which the real pairs’ actual behaviors were compared with predictions of models based on the individuals’ behavior.

We conceptualized the pairing effect as a consensus process by which the two individuals’ preferences were aggregated to create the pair’s behavior through discussion^29,30^. Assuming that each participant had a personal preference about how to behave in PSG, we used their behaviors (attack, move, or untouched) in the 1-on-1 PSG as inputs to the 2-on-2 PSG (audio recordings also confirmed that all of the 61 pairs started discussion in the 2-on-2 PSG by stating how each person had decided in the 1-on-1 PSG). We then examined how those personal preferences were aggregated into the pair’s behavior using the following 5 models. These models posited different aggregation processes for resolving initial disagreements within a pair:

1. The more aggressive wins: the pair will display the more aggressive behavior. As the three response categories (attack, move, untouched) are ordered in terms of inclination to strike the opponent, the model predicts that an attack/move pair will adopt “attack”, an attack/untouched pair will adopt “attack”, and a move/untouched pair will adopt “move” as the pair-level behavior.
2. The more peaceful wins: the pair will display the more peaceful behavior. That is, this model predicts that an attack/move pair will adopt “move”, an attack/untouched pair will adopt “untouched”, and a move/untouched pair will adopt “untouched”.
3. The more extreme wins (but aggression wins when there are two opposing extremists): the intermediate preference (move) will always concede to the more extreme preference (attack, untouched). That is, an attack/move pair will adopt “attack” (which is more extreme than “move” as the middle category on the response continuum), a move/untouched pair will adopt “untouched” (which is more extreme than “move” on the response continuum) as the pair-level behavior. An attack/untouched pair, where both members are extremists, will adopt “attack”.
4. The more extreme wins (but peace wins when there are two opposing extremists): the intermediate preference (move) will concede to the more extreme preference (i.e., an attack/move pair will adopt “attack”; a move/untouched pair will adopt “untouched”). But an attack/untouched pair, where both members are extremists, will adopt “untouched”.
5. Random: the pair will choose randomly between the two initial responses.

We first generated all possible nominal pairs (other than the real pairs) for each treatment and sex. Collapsed across the treatments and sexes, the total number of nominal pairs was 1,748 (=_28_C_2_ + _30_C_2_ + _30_C_2_ + _34_C_2_ − 61). Applying each of the 5 models to the nominal pairs, we derived predicted frequencies of the three response categories (attack, move, untouched) at the pair level (for details, see Supplementary Tables S3 and S4). For the Random model, we sampled 5,000 decisions randomly for each nominal pair to calculate mean frequencies for the three response categories at the pair level. The 2nd column of Table 1 summarizes overall results of the goodness-of-fit tests of the 5 models in terms of chi-square statistics. Among the 5 models, only “The more extreme wins (but peace wins when there are two opposing extremists)” model was not rejected (*χ*^2^ = 1.004, *df* = 2, *p* = 0.605), approximating the observed pair behaviors relatively well. Also, as seen in the 3rd and 4th columns of Table 1, this model was the only model that survived separate tests for the Saline condition and for the AVP condition simultaneously (Saline: *χ*^2^ = 2.775, *df* = 2, *p* = 0.250; AVP: *χ*^2^ = 1.550, *df* = 2, *p* = 0.461). These results mean that the more extreme preferences on the response continuum (i.e., attack, untouched) tended to dominate the less extreme (i.e., move) preference eventually at the pair-level behavior (but peace still won when the pair was composed of two opposing extremists), whether under AVP or not.

**Table 1:** Goodness-of-fit tests of the five pair-choice aggregation models in terms of chi-square statistics.

Model	Overall		Saline		AVP	
	χ^2^	*p*	χ^2^	*p*	χ^2^	*p*
More aggressive wins	138.230	<0.001	94.544	<0.001	46.441	<0.001
More peaceful wins	6.651	0.036	3.441	0.179	9.219	0.010
More extreme wins (with aggression advantage)	18.198	<0.001	13.038	0.001	8.019	0.0181
More extreme wins (with peace advantage)	1.004	0.605	2.775	0.250	1.550	0.461
Random	8.676	0.013	5.292	0.071	4.098	0.129

Note. To assess each model’s goodness of fit to the observed frequencies of pair choices, we conducted a chi-square test in a two-way cross-table with the model/observed × the response categories (attack, move, untouched: see Supplementary Tables S3 and S4 for the frequencies/percentages predicted by each model). Here, the 2nd column shows results of the model testing with the choice frequencies collapsed over treatments (saline vs. AVP) and sexes. The 3rd and 4th column reports a separate test for the saline and AVP condition respectively with the choice frequencies collapsed over sexes. The “more extreme wins (with peace advantage)” model was the only one that was not rejected (i.e., approximated the observed pair behavior reasonably well). In all rows, the *df* was 2.

## Discussion

Although accumulating evidence suggests that AVP is involved in territorial defense by non-human animals^9,10^, the evidence for this relation in humans has been rather scarce. In this study, we investigated the effect of intranasal AVP using an incentivized economic game (preemptive strike game^24^) that captures anxieties associated with resource defense in an interactive context. In both the 1-on-1 and 2-on-2 situations, participants’ aggressive choices were enhanced by AVP administration. Furthermore, this pattern was observed for both males and females. These results support our hypothesis that AVP generally enhances human defensive aggression against potential threats to their resources.

Several remarks can be made about these results from the individual-level analysis. First, we observed effects of exogenous AVP on preemptive strike, but found no significant relation between basal (pre-administration) urinary AVP and participant’s defensive aggression. The analysis of behavioral responses in the Saline condition yielded no effect of AVP level in pre-administration urine samples on defensive aggression (*β* = 0.005, *SE* = 0.007, *z* = 0.78, *p* = 0.440: see Supplementary Table S5). Likewise, the difference between pre- and post-administration urinary AVP had no effect on participant’s choice in the 1-on-1 PSG in the Saline condition (*β* = −0.004, *SE* = 0.008, *z* = 0.51, *p* = 0.610: Supplementary Table S6). These results indicate that only the exogenously-administered AVP affected participants’ behaviors in this experiment (see also Supplementary Tables S7 and S8). As shown in Supplementary Fig. S1, the changes in urinary AVP in the Saline condition were minimal, suggesting that the interactive context in this task may have been too weak or too short in duration to stimulate peripheral AVP secretion. Thus, the possible effects of endogenous AVP on human defensive aggression in longer interactions remain to be seen. Future studies that implement a task setting in which endogenous AVP changes dynamically through social interaction^30^ seem particularly promising to address this question.

Second, no major sex differences were detected in this study. Several previous human studies have reported some sex differences in the effect of intranasal AVP on perception of neutral faces. Thompson *et al*., for example, showed that AVP enhanced agonistic facial motor patterns in males, but affiliative facial motor patterns in females, in response to faces of unfamiliar persons of the same sex^3,4^. However, no such clear sex difference has been observed for cooperation rates in the Prisoner’s Dilemma game^18–21^. On the other hand, in hamsters, AVP enhanced aggression against unfamiliar intruders of the same sex in males, but inhibited aggression in females^31,32^, which accords with the abovementioned sex differences in human facial responses^3,4^. Given that AVP/AVT have multiple social functions including social recognition, social communication, and aggression (associated with mate competition, territory defense, or pup defense)^12,33,34^, it seems likely that sex differences depend on context for humans as well. However, the existing human studies, including ours, do not provide enough information on this critical issue. Future studies will benefit greatly by systematically examining possible sex differences in relation to different adaptive tasks that are crucial for survival.

Third, it could be argued that the attack in the PSG reflects spiteful behavior rather than defensive aggression. However, Simunovic *et al*., who developed the PSG, showed that the attack option was rarely chosen when the opponent had no capability of preemptive strike, indicating that the attack was fear-based and did not involve the spiteful goal of simply hurting the opponent^24^. In other words, the bilateral nature of PSG seems to be the key that promotes defensive aggression against potential threat^26^.

For the 2-on-2 PSG, the pairing procedure did not particularly promote aggressive shared decisions whether participants were under AVP or not (that is, the agonistic effect of AVP was not attenuated or enhanced by group discussion). Group-level enhancement in PSG was also not observed by Mifune *et al*.; they compared attack rates in 1-on-1 and 3-on-3 PSG behaviorally (without any hormone administration), but found no significant difference between the two conditions^35^.

The model-based analysis of the 2-on-2 game^29,30^ suggests that, in both the Saline and AVP treatments, “the more extreme wins (with peace advantage)” model approximated the pair behavior well: the pairing procedure generally reduced the influence of intermediate (Move) behavior against the more extreme positions (Attack or Untouched), while prioritizing peaceful behavior over aggressive behavior. While these results suggested some shifts toward the less aggressive behavior in consensus building, the effect of exogenous AVP remained marginally significant at the pair level. This suggests that intranasal AVP enhances individual-level propensity for aggression mainly, and that such effect has an enduring impact at the pair level.

There are some limitations of our study that should be addressed in future research. While we found some effects of exogenous AVP at the behavioral level, we were not able to address any underlying neural or physiological mechanisms in this study. Future research should investigate such processes more directly, including potential distinctions between AVP acting centrally or peripherally. Intranasal application can deliver AVP to both cerebrospinal fluid and the peripheral bloodstream^36^. Comparison experiments between intranasal administration and peripheral injection are necessary to distinguish AVP targets more clearly. Furthermore, dosage may impact the effect of AVP; a recent study reported that when 20 IU was administered intranasally, male participants gave more negative subjective ratings to same- or other-sex faces, relative to placebo and 40 IU^37^. While we used 40 IU here following the protocol of a recent game experiment^17^, systematic manipulation of dosage may shed light on boundary conditions of the agonistic or affiliative effects of AVP.

Overall, our study clearly illustrates a role for AVP in human defensive aggression during an experience of potential resource threat. As far as we know, this is the first study that has provided direct evidence for the effect of exogenous AVP on human defensive aggression. Neuropeptides such as AVP and Oxytocin (OT) have been linked with a wide range of social behavior both in animals and in humans^34,38–40^. For example, arginine vasotocin, a homolog of vasopressin, regulates territorial grunting vocalization in male fish^41^. In rats, vasopressin acts in the olfactory bulb to enable social recognition^42^. In monogamous voles, vasopressin is responsible for pair-bonding^43^ and territorial aggression to protect the nest area^9^. These animal results endorse the idea that vasopressin, which serves basic sexual and protective functions, can be coopted to regulate more complicated social behaviors in species that live in large, complex groups, like humans and monkeys. Although parallel evidence about humans is still elusive, these earlier studies suggest that neuropeptides may deeply regulate human social behavior as well. In this sense, investigating those processes will be essential toward better understanding and possibly regulating various social phenomena in our societies.

## Methods

### Participants

A total of 122 university students (58 males and 64 females; age 18–35, mean = 21.68 years, SD = 2.29) were summoned to participate in the experiment. They were required to refrain from taking any food or drink (other than water) for 2 hours before the session started. All sessions were run between 3 P.M. and 6 P.M. on weekdays. Upon arrival, all participants completed a prescreening questionnaire to determine whether they had medical problems (e.g., heart disease, kidney disease, migraine, allergies) or were pregnant. We also measured each participant’s blood pressure to confirm that it was below 135/85 mmHg. No participants were excluded by this prescreening. We randomly assigned all participants in a session as a group to either the AVP or Saline treatment. As a result, there were 58 participants (30 females) in the AVP condition and 64 participants (34 females) in the Saline condition. The study was approved by and carried out in accordance with the guidelines and regulations of the ethical committee of the Department of Social Psychology of the University of Tokyo. All participants gave written informed consent, approved by the ethical committee, prior to the experiment. After the experiment, participants received compensation (mean = 5911.5 JPY [=52 USD], SD = 936.5) for their participation in the experiment.

### Materials

Intranasal AVP—0.6 ml of 40 IU AVP (concentrated by water evaporation) was transferred to a plastic nasal spray bottle (Pitressin Injection, Dai-ichi-Sankyo. Pharm. Co. Ltd.).

Intranasal placebo—0.6 ml of saline solution was transferred to a plastic nasal spray bottle. In the post-session questionnaire, participants were asked which treatment (saline or AVP) they thought they had received. As shown in Supplementary Tables S9 and S10, proportions of correct responses were not significantly different from chance in either treatment by a binomial test (Saline: *p* = 0.512; AVP: *p* = 0.314).

### Procedure

Four same-sex participants who had not met before the experiment were called to each session. In 7 (of the 35 total) sessions, only two participants showed up, so we added two same-sex confederates as explained below. Upon arrival, each participant was seated in a private soundproof cubicle. They were informed that all participants in the session were of the same sex, but no communication was allowed with other participants at this point. Participants were then informed about the experimental procedure and possible side effects of AVP. It was emphasized that participants could leave the experiment with compensation at any time for any reason. If participants agreed to participate in the experiment, they were asked to answer the prescreening questionnaire. Their blood pressure was also measured. Each participant then left the laboratory for pre-administration urine sampling.

After coming back to the laboratory, participants were asked to self-administer AVP or saline intranasally. All participants in a session received the same (AVP or saline) treatment. They were instructed to place the nasal applicator in one nostril and to squeeze the pump while inhaling the spray mist until they ran out of solution. We confirmed that each participant emptied the spray bottle and completed the nasal administration. Following previous studies^15,17^, we included a 30-minute rest period here, so that participants would perform the game task during the time window when the level of neuropeptides in the cerebrospinal fluid had reached its peak (30–60 minutes after intranasal AVP administration)^36^.

After the game task was completed, participants individually answered a post-session questionnaire. The second urine sample (post-administration) was collected after the questionnaire (see Supplementary Fig. S1 for the pre-post administration difference in creatinine-adjusted urinary AVP concentration). The timing of this sampling was at least 90 minutes after intranasal administration, which is known to be sufficient for the excretion of intranasally-administered neuropeptide in urine^44^. Participants were then debriefed, paid, and dismissed.

### Preemptive strike game

Thirty minutes after the AVP or saline administration, participants were given instructions for the PSG in the private cubicles. The instructions were spoken aloud to participants by a pre-recorded artificial voice. While listening to the spoken instructions, each participant also read the written version on a tablet. As in previous studies^24,27^, we carefully avoided using loaded words (“preemptive strike”, “attack”, “defense”, etc.) in the instructions or on the computer screen. We provided only factual, neutral explanations of the procedure for the experiment.

Besides a 3,500 JPY (about 31 USD) show-up fee, we provided an additional 3,000 JPY to participants as an endowment for the PSG. During the game, participants had 15 seconds during which they decided whether to take no action or to engage in a preemptive strike (though we did not use these words in the experiment) by incurring a cost deducted from their endowment. Figure 1 shows the game interface that each participant saw on a desktop computer screen placed about 45 cm away in front of each participant. Participants had to choose: (1) to take no action during the 15 seconds, or (2) to move the mouse pointer to click a red button displayed on the center of the screen. If both players chose to take no action during the 15 seconds, each player kept their initial endowment intact, receiving 3,000 JPY. Otherwise, the player who clicked the button first would incur a 700 JPY cost (and thus receive a net 2,300 JPY), and the other player who took no action during the 15 seconds or clicked the button later than their counterpart would lose 2,500 JPY (and thus receive 500 JPY). Participants were told that they would be paid according to the game outcome plus the fixed show-up fee at the end of the experiment.

As shown in Fig. 1, participants operated the mouse to choose their option. Participants were instructed that a mouse cursor would appear in the bottom part of the computer display a few seconds after the game started, and that this timing would be identical for both players in the PSG. For each game, the initial delay in the mouse appearance was randomly determined from a uniform distribution ranging from 2 to 4 seconds. Thus, participants were not able to precisely predict when they could start operating the mouse.

After the instructions, participants first played the PSG just once in a 1-on-1 (individual vs. individual) situation. They were told that there were 8 participants in the laboratory and that they would be matched with another participant randomly. In actuality, there were only 4 participants in each of the 28 sessions (and 2 participants + 2 confederates in the 7 sessions). In the 1-on-1 situation, each player was given 3 minutes after the instructions to think about how they would play before the game interface (Fig. 1) appeared.

Without providing feedback about the outcome from the 1-on-1 situation, the experiment proceeded to the second stage, in which participants played the PSG just once in a 2-on-2 (pair vs. pair) situation. Participants were instructed that they would be paired with another participant as a team and play the PSG against another team (pair). In order to prevent possible carryover effects from playing against the same previous opponent, we told participants that their team-partner and the opponent pair in the 2-on-2 situation would all be different from their opponent in the 1-on-1 situation. Logically, this would have required us to have 8 participants in a single session. However, because we were concerned about the logistical difficulty of administering AVP to 8 participants simultaneously and supervising their behavior, we decided to employ a minor deception (“8 participants”, “brand new opponents”) while actually running only 4 participants per session. In the 7 sessions with only two participants, a pair from a prior session was selected randomly as their opponent team to determine the game payoff for compensation after the experiment. In the 1-on-1 situation, each participant’s choice was randomly pitted against a choice by one of the two participants in the prior-session pair. In the 2-on-2 situation, the two participants’ choice as a pair was pitted against the prior-session pair’s choice.

In the 2-on-2 situation, each pair was given 3 minutes to become acquainted with each other and another 3 minutes to discuss how to play the game as a team before the game interface (Fig. 1) appeared. Participants were instructed that one member should operate the mouse as a representative of the pair, and that the two players in the same team would each receive the same payoff from the team choice. The game duration (15 seconds) was identical to that in the 1-on-1 situation.

## Supplementary information

Supplementary Information

## Data Availability

The materials and datasets used in the current study are available from the corresponding author on reasonable request.
