# Supplementary material for: Vasopressin enhances human preemptive strike in both males and females
Source: Sci Rep. 2019 Jul 4;9:9664. doi: 10.1038/s41598-019-45953-y (PMC6609689; doi:10.1038/s41598-019-45953-y)
Supplement: Supplementary file 1 — Supplementary Information [file 41598_2019_45953_MOESM1_ESM.pdf]

SUPPLEMENTARY INFORMATION

Vasopressin enhances human preemptive strike in both males and females

Atsushi Kawada[1], Miho Nagasawa[2], Aiko Murata[3,4], Kazutaka Mogi[2], Katsumi Watanabe[4,5], Takefumi Kikusui[2], Tatsuya Kameda[1,6,7] *

[1]*Department of Social Psychology, The University of Tokyo, Tokyo, Japan*

[2]*Department of Animal Science and Biotechnology, Azabu University, Kanagawa, Japan*

[3]*NTT Communication Science Laboratories, NTT Corporation, Kanagawa, Japan*

[4]*Faculty of Science and Engineering, Waseda University, Tokyo, Japan*

[5]*Creative Robotics Lab, University of New South Wales, Sydney, Australia*

[6]*Brain Science Institute, Tamagawa University, Tokyo, Japan*

[7]*Center for Experimental Research in Social Sciences, Hokkaido University, Hokkaido, Japan*

*Direct correspondence to:

Tatsuya Kameda, Ph.D. (tkameda@l.u-tokyo.ac.jp)

Department of Social Psychology, The University of Tokyo

7-3-1 Hongo, Bunkyo-ku, Tokyo 113-0033, Japan

Tel: +81-3-5841-3870, Fax: +81-3-3815-6673

Urinary sampling procedure

For both pre- and post-administration sampling, each participant put their own urine into a 15 ml plastic tube in a toilet room. The samples were stored in a cooler box with crushed ice until they were transferred to a refrigerated centrifuge maintained at 4 °C. After the urine samples were centrifuged, they were stored in a -80 °C deep freezer until the urinary AVP (U-AVP) concentrations were measured. For the U-AVP measurement, we used a commercially available kit from Enzo Life Sciences (ADI-900-017A). Below, U-AVP concentrations are expressed as the vasopressin-to-creatinine ratio.

A group difference in pre-post administration urinary AVP

The pre-post administration difference of U-AVP was larger in the AVP condition than in the Saline condition (*Brunner-Munzel Test Statistic* = 16.847, *df* = 70.853, *p* = 0.0001). This confirmed that the experimental treatment was successful.

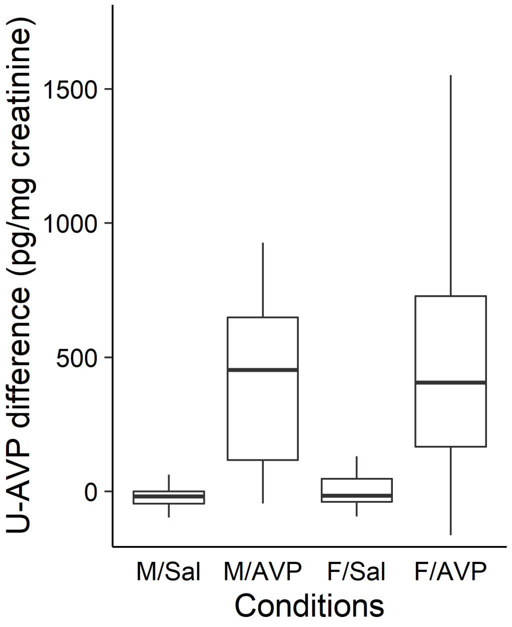

Supplementary Figure S1. The pre-post administration difference in creatinine-adjusted urinary AVP concentration as a function of the treatment and sex

Model selection for proportional-odds ordinal logistic regression of participants' choices using Akaike Information Criterion (AIC)

In both the 1-on-1 (Supplementary Table S1) and the 2-on-2 (Supplementary Table S2) PSG, the model with only a main effect of Treatment was best in terms of AIC.

Supplementary Table S1. Results from the 1-on-1 PSG

Model	*k*	log*L*	AIC
Null	2	-127.01	258.01
Sex	3	-127.00	260.00
Treatment	3	-123.51	253.01
Sex + Treatment	4	-123.50	255.01
Sex + Treatment + Sex × Treatment	5	-123.39	256.78

Note. *k:* the number of estimated parameters. log*L*: the maximum log likelihood. AIC: Akaike Information Criterion.

Supplementary Table S2. Results from the 2-on-2 PSG

Model	*k*	log*L*	AIC
null	2	-52.05	108.10
Sex	3	-51.99	109.99
Treatment	3	-50.21	106.43
Sex + Treatment	4	-50.12	108.23
Sex + Treatment +Sex × Treatment	5	-50.11	110.22

Note. *k:* the number of estimated parameters. log*L*: the maximum log likelihood. AIC: Akaike Information Criterion.

Model-based analysis of pair choices (see text for explanations of the models)

"The more extreme wins (but peace wins when there are two opposing extremists)" model provided the best fit to the overall frequencies (collapsed across both the treatment and sex: Supplementary Table S3), as well as to the frequencies in the Saline and AVP condition respectively (collapsed across sex: Supplementary Table S4).

Supplementary Table S3. Observed and predicted overall frequencies (proportions) of the three response categories at the pair level (collapsed across treatment and sex)

Model	Untouched	Move	Attack
Real (observed) behavior	41 (.672)	8 (.131)	12 (.197)
The more aggressive wins	226 (.130)	721 (.412)	801 (.458)
The more peaceful wins	1084 (.620)	451 (.258)	213 (.122)
The more extreme wins (with aggression advantage)	725 (.415)	222 (.127)	801 (.458)
The more extreme wins (with peace advantage)	1084 (.620)	222 (.127)	442 (.253)
Random	870.12 (.498)	499.01 (.285)	378.87 (.217)

Note. Numbers in parentheses refer to proportions in each row.

Supplementary Table S4. Observed and predicted frequencies (proportions) of the three response categories at the pair level in the Saline and AVP treatments (collapsed across sex)

Model	Saline			AVP		
	Untouched	Move	Attack	Untouched	Move	Attack
Real (observed) behavior	25 (.781)	3 (.094)	4 (.125)	16 (.552)	5 (.172)	8 (.276)
The more aggressive wins	135 (.140)	453 (.470)	376 (.390)	91 (.116)	268 (.342)	425 (.542)
The more peaceful wins	615 (.638)	217 (.225)	132 (.137)	469 (.598)	234 (.298)	81 (.103)
The more extreme wins (with aggression advantage)	445 (.462)	143 (.148)	376 (.390)	280 (.357)	79 (.101)	425 (.542)
The more extreme wins (with peace advantage)	615 (.638)	143 (.148)	206 (.124)	469 (.598)	79 (.101)	236 (.301)
Random	574.06 (.596)	254.01 (.263)	135.93 (.141)	296.06 (.378)	245.00 (.313)	242.94 (.310)

Note. Numbers in parentheses refer to proportions in each row in each condition.

Analysis of effect of basal urinary AVP (in the pre-administration urine samples) on defensive aggression in the Saline condition

The proportional-odds ordinal logistic regression revealed no significant effect of basal U-AVP on defensive aggression in the 1-on-1 PSG in the Saline condition (n = 64).

Supplementary Table S5. Results of the regression analysis with participant's basal (pre-administration) U-AVP, sex, and interaction as predictors

Predictor	Parameter Estimates	Standard Error	z	p
Basal U-AVP	0.0053242	0.0068594	0.776	0.438
Sex (0 = Male, 1 = Female)	-0.2693170	1.0836936	0.249	0.804
Basal U-AVP × Sex	0.0008679	0.0078154	0.111	0.912
Log-Likelihood	-58.32			
AIC	126.63			

Analysis of effect of pre-post administration difference of U-AVP on defensive aggression

The ordered logistic regression model revealed no significant effect of pre-post administration U-AVP difference (post – pre) on participant's choice in 1-on-1 PSG in the Saline condition (Supplementary Table S6). This was also the case in the AVP condition (Supplementary Table S7) as well as for the overall data collapsed across the two conditions (Supplementary Table S8).

Supplementary Table S6

Results of the regression analysis with pre-post administration U-AVP difference (post – pre), sex and interaction as predictors in the Saline condition

Predictor	Parameter Estimates	Standard Error	*z*	*p*
U-AVP pre-post difference	-0.004272	0.008434	0.507	0.613
Sex (0 = Male, 1 = Female)	0.045802	0.530897	0.086	0.931
U-AVP pre-post difference × Sex	0.001442	0.009576	0.151	0.880
Log-Likelihood	-57.66			
AIC	125.31			

Supplementary Table S7

Results of the regression analysis with pre-post administration U-AVP difference (post – pre), sex and interaction as predictors in the AVP condition

Predictor	Parameter Estimates	Standard Error	*z*	*p*
U-AVP pre-post difference	0.0000494	0.0009712	0.051	0.959
Sex (0 = Male, 1 = Female)	0.8910763	0.7701345	1.157	0.247
U-AVP pre-post difference × Sex	-0.0013288	0.0012389	1.073	0.283
Log-Likelihood	-60.69			
AIC	131.38			

Supplementary Table S8

Results of the regression analysis with treatment, pre-post administration U-AVP difference (post – pre), and sex as predictors for the overall data collapsed across the two conditions

Predictor	Parameter Estimates	Standard Error	*z*	*p*
Treatment (0 = Saline, 1 = AVP)	1.4000430	0.4648591	3.012	0.0026
U-AVP pre-post difference	-0.0008759	0.0006053	1.447	0.1479
Sex (0 = Male, 1 = Female)	0.1332135	0.3548754	0.375	0.7074
Log-Likelihood	-119.32			
AIC	248.65			

Analysis of participants' awareness of treatment condition

In the post-session questionnaire, participants were asked which treatment (saline or AVP) they thought they had received. Supplementary Table S9 displays frequencies (and proportions) of the correct and incorrect responses in each treatment. As shown in Supplementary Table S10, the proportions of correct responses were not significantly different from the chance level by a binomial test (Saline: $p = 0.512$; AVP: $p = 0.314$). A chi-square test also showed that the correct frequencies were not distinguishable between the two treatments, χ^2 (1) = 1.3734, $p = 0.241$.

Supplementary Table S9

The frequencies (and proportions) of participants who correctly or incorrectly answered the question "which treatment do you think you received?"

Treatment	Correct	Incorrect
Saline	32 (0.552)	26 (0.448)
AVP	27 (0.429)	36 (0.571)

Note. One participant in the AVP condition failed to complete the questionnaire.

Supplementary Table S10

Results of binomial tests of the correct rates against the chance level (0.50)

Treatment	Responses	*n*	Observed Prop.	Test Prop.	*p*
Saline	Correct	32	0.552	0.500	0.512
	Incorrect	26	0.448		
AVP	Correct	27	0.429	0.500	0.314
	Incorrect	36	0.571		
